# Diagnosis of HEV infection by serological and real-time PCR assays: a study on acute non-A-C hepatitis collected from 2004 to 2010 in Italy

**DOI:** 10.1186/1756-0500-5-297

**Published:** 2012-06-15

**Authors:** Angela Candido, Stefania Taffon, Paola Chionne, Giulio Pisani, Elisabetta Madonna, Stefano Dettori, Abir Hamza, Catia Valdarchi, Roberto Bruni, Anna Rita Ciccaglione

**Affiliations:** 1 Department of Infectious, Parasitic and Immune-Mediated Diseases, Istituto Superiore di Sanità, Rome, Italy; 2 Center for Immunobiologicals Research and Evaluation, Istituto Superiore di Sanità, Rome, Italy; 3 Laboratory of Transmissible Illness and Active Biological Substances, Faculty of Pharmacy, University of Monastir, Monastir, Tunisia

## Abstract

**Background:**

The impact of hepatitis E in developed countries, like Italy, still requires a clear definition. In the present study, we evaluated HEV infection in patients with acute non-A-C hepatitis by an approach comparing data from Real-time PCR and serological assays.

**Methods:**

In a first analysis, sera from 52 patients hospitalized with a diagnosis of acute viral non-A-C hepatitis in Italy were tested by in-house Real-Time PCR assay for identification of Hepatitis E Virus (HEV) RNA and by anti-HEV IgM and IgG assays. In a subsequent analysis, selected samples were evaluated by additional IgM tests to confirm diagnosis.

**Results:**

Among the 52 samples, 21 showed positive results for all three markers (IgM, IgG and HEV RNA). One patient showed HEV RNA as single marker. Uncertain results were found in 8 samples while the remaining 22 were negative for all markers. Further analysis of the 8 undefined samples by additional IgM tests confirmed HEV infection in 1 patient. Overall, acute HEV infections were reliably identified in 23 (44.2%) out of 52 patients.

**Conclusions:**

In the present paper, we performed a study evaluating HEV infection in 52 sporadic non-A-C acute hepatitis cases. All samples were collected from 2004 to 2010 in Italy. By a diagnostic strategy based on genomic and serological assays we identified HEV infections in 23 out of 52 patients (44.2%), a percentage higher than previous estimates. Thus, the actual impact of HEV infections in Italy needs to be further evaluated on a national scale by a diagnostic strategy based on multiple and last generation assays.

## Background

Hepatitis E virus (HEV) is the major cause of several outbreaks of water-borne hepatitis in countries with poor sanitation and of sporadic cases of acute viral hepatitis in endemic and industrialized countries. In the latter’s the disease was initially found to occur almost exclusively among immigrants or travelers returning from endemic areas. However, over the last one decade, identification and characterization of swine HEV in the United States, Europe and many other countries as well as their close relationship with human HEV found in the same geographic areas prove that HEV is indeed a zoonotic virus and that domestic swine, wild deer and boars are reservoirs of HEV in nature [1-5].

Hepatitis E is caused by a non-enveloped, single-stranded, positive sense RNA virus that is the only member of the genus *Hepevirus* in the family *Hepeviridae*. HEV sequences have been classified into four genotypes divided into several subtypes. While HEV genotype 1 is hyper-endemic in Asia and Africa, where it causes outbreaks and sporadic acute hepatitis, HEV genotype 3 is prevalent in developed nations, where sporadic acute hepatitis due to this virus was identified [6,7].

An increasingly number of autochthonous cases has been recently reported in Western Europe, especially in the UK [8,9], the Netherlands [10], France [11,12] and Spain [13] suggesting that hepatitis E is an emerging disease in developed countries.

Diagnosis of HEV acute cases is based on detection of anti-HEV IgM and/or HEV RNA in sera. However, this field is constantly evolving as performance of serological tests in some cases is not optimal and genomic tests with increased sensitivity were developed in the last years. Sensitivity of currently available commercial assays for IgM is usually within 91-97% [14-16], but they may produce false negative results in genotype 1 infected patients [14] or false positive results particularly in patients with IgM-rheumatoid factors in the serum [17] or suffering the acute primary infection by human cytomegalovirus (CMV) and Epstein-Barr virus (EBV) [18]. Positivity to HEV RNA in serum represents a reliable marker of ongoing infection. However, the onset, duration and levels of viremia should be defined more rigorously in order to optimize the diagnostic approach. Indeed, HEV RNA level was always analyzed by nested PCR genomic tests [13,19-23] which showed a lower sensitivity than Real-Time PCR methods recently developed [24].

In Italy, data from the national surveillance system for acute hepatitis indicated that HEV is responsible for nearly 10% of the acute cases [25]. This percentage is an underestimation as systematic testing of non-A-C cases for hepatitis E is not routinely performed in the infectious disease units. A long term prospective study from Italy, conducted over 15 years, revealed that 20.6% of non-A-C patients had acute HEV infection. Most cases (83.6%) were imported and caused by genotype 1 while some autochthonous cases were caused by genotype 3 [19]. However, the clinical impact of hepatitis E in Italy still remains to be clarified as the increased sensitivity and specificity of the last generation assays suggest a reassessment of previous percentages.

In the present work, we performed a study on 52 subjects hospitalized in Italy with a diagnosis of acute viral non-A-C hepatitis. HEV diagnosis was addressed by an approach comparing data from Real-time RT-PCR and current serological assays. Serum samples were collected in 17 infectious disease units, situated in 11 of the 20 regions of Italy, from 2004 to 2010.

## Results

### Screening of samples by serological and genomic assays

Fifty-two serum samples were first tested with two assays containing recombinant HEV antigens designed for detection of HEV IgM and IgG antibodies: the Bioelisa HEV IgM and IgG kits, Biokit. Table 1 shows results of genomic and serological assays for 30 out of 52 patients. Negative results for all three markers were obtained for the remaining 22 patients (data not shown).

**Table 1:** Positive results in serological and real-time PCR assays are shown in bold

**Patient code**	**Results of serological assays **^***a***^		**Results of real-time PCR (HEV-RNA **^***b***^**)**	**HEV genotype**	Country of origin *	**ALT (UI/L)**
	**Bioelisa HEV - IgM**	**Bioelisa HEV - IgG**				
8	>**3.000**	**>3.000**	+ + +	1a	Italy (India)	2640
3	**1.304**	**>3.000**	+ + +	1a	Bangladesh	1726
12	>**3.000**	**>3.000**	+ + +		India	1072
39	**0.882**	**>3.000**	+ + +		India	2463
16	**1.801**	**>3.000**	+ +	1a	Bangladesh	1263
19	**1.695**	**>3.000**	+ +		Bangladesh	835
22	>**3.000**	**>3.000**	+ +	1a	Italy (India)	n.a.
43	**0.508**	**>3.000**	+ +		Bangladesh	4515
47	**0.867**	**>3.000**	+ +		Bangladesh	1683
48	**2.709**	**>3.000**	+ +		Bangladesh	2050
52	**0.869**	**>3.000**	+ +	1a	Bangladesh	2496
55	**1.316**	**>3.000**	+ +		India	1263
56	**1.508**	**>3.000**	+ +		Bangladesh	n.a.
58	**0.793**	**2.070**	+ +		Italy (Thailand)	4044
60	**2.665**	**0.775**	+ +	3e	Italy	2010
7	**0.498**	**>3.000**	+ +		India	486
51	>**3.000**	**>3.000**	+ +		Italy	n.a.
61	**0.630**	**1.972**	+ +	1a	Italy (India)	2472
10	**2.388**	**>3.000**	+		Ethiopia	5328
29	**2.212**	**>3.000**	+		Bangladesh	n.a.
34	>**3.000**	**2.969**	+		Italy	855
63	0.102	0.088	+ + +		Albania	597
33°	0.057	**1.354**	+		Italy	384
20°	**0.653**	0.151	-		Italy (Africa)	over
30°	**2.986**	0.336	-		Pakistan	n.a.
50°	**0.480**	0.081	-		Italy	3860
67°	**0.546**	0.000	-		Italy (Africa)	842
26°	0.271	**0.814**	-		n.a.	2050
28°	0.062	**0.709**	-		Bangladesh	806
32°	0.126	**1.226**	-		Italy	2391

^*a*^ Bioelisa HEV IgM, (cut-off = 0.421); Bioelisa HEV IgG, (cut-off = 0.574); Biokit.

^*b*^ +: 250 < copies/mL < 2,500; + +: 2,500 < copies/mL <25,000; + + +: >25,000 copies/mL; -: < 250 copies/mL.

^*^ All foreign patients either arrived in Italy or travelled to their country of origin less than one month before the onset of clinical symptoms. Travel to countries from endemic areas are indicated in brackets for Italian patients.

n.a. : not available.

°Serum was also analysed with HEV IgM Elisa MP Diagnostics (cut-off = 0.403); HEV IgM Elisa Wantai Biopharm (cut-off = 0.263)**.** All samples were negative except sample 33 which showed OD values of 0.421 and 0.334 in MP Diagnostic and Wantai Biopharm assays, respectively.

As shown in Table 1, IgM and/or IgG antibodies were detected in 29 out of 52 patients (55.7%). Among the 29 positive samples, 21 (72.4%) showed both IgM and IgG antibodies, while 4 (13.7%) samples showed IgM only (Table 1, patients 20, 30, 50 and 67) and 4 (13.7%) samples only IgG (Table 1, patients 26, 28, 32 and 33). HEV RNA was found in 23 out of 52 samples (44.2%).

### Diagnosis of HEV acute infection by serological and genomic markers

To better define acute infections, we analyzed overall results from all three diagnostic assays. Twenty-one out of 52 samples (40.3%) showed positive results for all three markers (IgM, IgG and HEV RNA) (Table 1, the first 21 patients). HEV RNA was also detected in 1 sample not identified by the IgM and IgG assays (Table 1, patient 63). At least for these patients, a reliable confirmation of HEV ongoing infections was obtained.

The last 8 samples listed in Table 1 presented uncertain results. To better define diagnosis, we analyzed these samples with other IgM commercial assays: the HEV IgM Elisa MP Diagnostics and the HEV IgM Elisa Wantai Biopharm. In addition, CMV and EBV infection markers were evaluated by the assays: anti-EBV VCA IgM Elisa, Bio-Rad and Bioelisa CMV IgM, Biokit. In a first analysis, patient 33 had shown HEV RNA and IgG positivity but it was negative for IgM antibodies by the Bioelisa assay (Table 1). Additional IgM assays confirmed HEV acute infection: in fact, the sample showed OD values higher than cut-off (0,421 and 0.334 in MP Diagnostic and Wantai Biopharm assays respectively; see footnote in Table 1 for cut-off).

Four samples had shown only IgM antibodies in the first analysis (Table 1, patients 20, 30, 50 and 67). These data were not confirmed by the other IgM assays (data not shown) suggesting they were false positive results. The last 3 patients had shown only IgG antibodies (Table 1, patients 26, 28 and 32). This serological profile, which indicates a past HEV infection, was confirmed by further analysis (data not shown).

All 8 samples were negative for anti-EBV IgM; only patient 30 showed anti-CMV IgM positivity (data not shown).

Overall, HEV acute infections were reliably detected in 23 out of 52 patients (44,2%) (Table 1, the first 23 patients).

### Genotyping of HEV isolates

Among the 23 viremic patients, 19 (15 foreigners and 4 Italians) developed hepatitis after returning from a travel to endemic areas (Table 1). Phylogenetic analysis confirmed infection with HEV of genotype 1a for 6 of them (Table 1, patients 3, 8, 16, 22, 52, and 61). Analysis of HEV sequence obtained from one of 4 patients, who did not travel abroad, confirmed infection with HEV of genotype 3e (Table 1, patient 60).

## Discussion

In the present paper, we performed a study on sporadic cases of acute non-A-C hepatitis collected in Italy by an approach comparing data from Real-time RT-PCR and current serological assays.

Acute HEV infections were reliably identified in 23 out of 52 patients (44.2%). For 22 of them, infection was confirmed by detection of all three markers. Thus, in agreement with the dynamic of acute infection, most of patients (22 out of 23) were in the early post-seroconversion stage (all three markers positive); only 1 patient was in the window period of acute phase where antibodies were not yet detectable and viremia was the only marker of infection.

Analysis of quantitative data showed that 82.6% of patients (19 out of 23) presents a medium to high level of viremia (2,500 < copies/mL < over 25,000) while only 17.3% (4 out of 23) has lower levels (250 to 2,500 copies/mL). These findings suggest majority of patients at presentation could be easily diagnosed by a genomic test with a minimal risk of false negative PCR results.

The problem of false positive or false negative results in IgM testing is a critical point in HEV diagnosis [14-18]. As evidence of this, patient 33 had shown a serological pattern characterized by the presence of HEV RNA and IgG antibodies. Such unusual pattern was due to a false negative result in the IgM test. In fact, additional testing by other commercial IgM assays helped to define a typical acute case with positivity to all three markers. Similarly, four patients (20, 30, 50 and 67) had shown IgM antibodies as single serological marker at first analysis, a result not confirmed by other assays, supporting false positive HEV IgM results. At least in one case (patient 30) false positivity was likely due to polyclonal B-cell stimulation caused by acute primary CMV infection [18]. Present findings reinforce previous warning about HEV diagnosis based on IgM marker only (as clear examples, see results from patients 30, 33 and 63) and stress the need to combine complementary methods and/or eventually confirm the results by additional IgM tests.

In Italy, data from the national surveillance system for acute hepatitis indicated that HEV is responsible for nearly 10% of the acute cases [25]. Interestingly, data reported from a series of 277 cases studied in Spain, with 30 cases of acute HEV diagnosed in total, gave an almost identical percentage (11%) [13]. This suggests that, in a comparison between all European countries, Italy could be close to Spain for the circulation of HEV. This interesting observation, however, still requires further study as the national surveillance system for acute hepatitis also warns about an underestimation of cases in Italy [25].

In a recent paper, HEV acute infections were identified in 20.6% of non-A-C hepatitis patients in Italy [19]. Most cases (83.6%) were imported and caused by genotype 1 while some autochthonous cases were caused by genotype 3. In agreement with this study, most of our acute cases are imported (19 out of 23, 82.6%), but a higher percentage of HEV infections on the total number of non-A-C acute cases was observed (44.2% *vs.* 20.6%). Although the previous study was conducted on a much larger number of patients than our, samples were collected and tested during the study-time (1994–2009) using different in-house and commercially available immunoassays, with variable sensitivity and specificity over time [19]. In our study, all samples were tested by the same set of diagnostics, exclusively including current last generation assays: this approach likely could explain the higher percentage of HEV cases.

Despite the limited number of samples, the present study raises a reasonable question about the actual number of HEV acute infections in Italy, suggesting it may be higher than previously estimated [19,25]. This issue is particularly important for Italy, which shows a high and increasing proportion of non-national population (7.5%, according to the most recent estimate in 2010) [26]. On this basis, a national large scale study to exactly evaluate the impact of HEV acute infections in Italy is needed.

## Conclusions

In the present paper, we performed a study evaluating HEV infection in 52 sporadic non-A-C acute hepatitis cases. All samples were collected from 2004 to 2010 in Italy. A diagnostic strategy based on current genomic and serological assays was applied. Acute HEV infections were reliably identified in 23 out of 52 patients (44.2%), a percentage higher than previous estimates. Thus, the actual impact of HEV infections in Italy still deserves further investigation on national scale.

## Methods

### Patient samples

From February 2004 to November 2010, serum samples from 52 patients with clinically suspected viral hepatitis were collected in 17 Italian infectious disease units in 11 of the 20 regions of Italy. Viral hepatitis was suspected in patients who presented anorexia, nausea, malaise, abdominal pain, dark urine, jaundice and scleral icterus and abnormal ALT levels. All patients were hospitalized with a diagnosis of acute viral non-A-C hepatitis based on serological and virological tests (serum samples negative for anti-HAV IgM, HBsAg, anti-HBc IgM, anti-HCV and HCV-RNA) and exclusion of autoimmunity, alcohol consumption or hepatotoxic drugs use. The samples were sent to the Viral Hepatitis Unit of the Italian National Institute of Health for diagnosis of HEV acute infection. Written informed consent for participation in the study was obtained from participants or, where participants are children, a parent or guardian. The study was approved by the Ethics Committee of the Italian National Institute of Health. The mean age of the 52 patients was 33 years, ranging from 2 to 78; 63,5% were males and 36,5% females; ALT values were available for 38 individuals, mean ALT peak value was 1868 IU/L, ranging from 200 to 6707 IU/L. As described in detail in the next paragraphs, sera were tested by (a) anti-HEV IgM assay (b) anti-HEV IgG assay, and (c) in-house Real-Time PCR assay for detection of HEV genomic RNA.

### Anti-HEV IgM and IgG assays

All assays were performed following the manufacturers’ package insert. All sera were tested using the following commercial kit: Bioelisa HEV IgM (cut-off = 0.421) and Bioelisa HEV IgG (cut-off = 0.574) (Biokit, Barcelona, Spain). Where indicated, sera were also tested using the following commercial assays: HEV IgM Elisa 3.0 (cut-off = 0.403) (MP diagnostics, MP Biomedicals Asia Pacific Pte. Ltd.-Singapore); HEV IgM Elisa (cut-off = 0.263) (Wantai Biopharm, Beijing Wantai Biological Pharmacy Enterprise CO., Ltd- China)**;** Anti-EBV VCA IgM ELISA (Bio-Rad Medical Diagnostics GmbH Dreieich, Germany); Bioelisa CMV IgM (Biokit, Barcelona, Spain).

### Viral RNA extraction and quantitative real-time PCR

HEV RNA was extracted from 200 μL of serum using silica columns provided with the QIAamp MinElute Virus Spin kit (Qiagen, Hilden, Germany) according to the manufacturer’s instructions. Every sample was spiked with four μL of RNA Internal Extraction Control (PrimerDesign Ltd, UK) added to AVL buffer at the extraction step. 60 μL of equivalent serum volume were amplified on Light-Cycler V2.0 using Quantitect RT PCR (Qiagen, Hilden, Germany) and specific HEV primers and TaqMan probe that recognize the ORF3 region. The specificity of the assay was previously evaluated by testing 22 viral (non-HEV) specimens [27].

All precautions to avoid PCR contamination were taken. The closed system for amplification and detection used with Real-Time PCR virtually eliminates the amplicon contamination caused by the opening and closing of reaction vessels that is a typical procedure for the conventional PCR and detection methods (to perform nested PCR or to run the amplified product on agarose gel). In addition, the PCR laboratory is divided in three separate rooms: one for reagent preparation, the second one for the extraction step and the third one for amplification and detection step. In order to obtain a further level of amplicon contamination control, in the amplification step the TTP base is substituted by UTP base and the uracil-N-glycosylase is added into the PCR mix. Finally negative controls (negative plasma and only PCR regent) were included in each run.

The ability of the Real-Time RT PCR assay to detect 100–250 copies/mL of HEV-RNA was investigated in the context of an Interlaboratory Study organized by the Paul-Erlich Institute [24]. In this study a panel comprising 22 HEV blinded positive serum samples representing tenfold serial dilutions of genotypes 3a, 3b, 3f and 4c was distributed to 20 laboratories from 10 different countries.

Three calibration samples were prepared using an HEV genotype 3b reference preparation at a final concentration of about 25,000, 2,500 and 250 copies/mL. In relation with the crossing point values observed for the three diluted calibration standard samples, it was possible to have a rapid estimate of the viral load expressed as follow: non-reactive or below the detection limit (−): < 250 copies/mL; reactive (+): 250 to 2,500 copies/mL; reactive (++): 2,500 to 25,000 copies/mL, and reactive (+++): over 25,000 copies/ml.

### Nested PCR amplification for sequencing

Viral RNA was extracted from 200 μl of serum as described in the previous paragraph. A 457 bp region (nt 5982–6438) of the ORF 2 of HEV genome was amplified by nested RT-PCR reaction, as described elsewhere [28]. A 10 μl RNA volume was reverse transcribed using 1 μl of 0.03 OD260/μl random primers and 200 U of Moloney murine leukemia virus reverse transcriptase (Invitrogen, Life Technologies, Carlsbad, CA) in a final volume of 20 μl at 50°C for 1 h, followed by 15 min at 70°C. One-half of the cDNA was used in PCR. After a denaturation step of 2 min at 94°C, DNA was amplified for 35 cycles at 94°C for 30 sec, 55°C for 30 sec, and 72°C for 1 min (and an additional 7 min at 72°C in the last cycle) in a final volume of 50 μl, containing 1X PCR buffer, 1.5 mM MgCl_2_, dNTP mixture (0.2 mM each), 100 pmol of each primer and 1U of Platinum Taq DNA polymerase (Invitrogen, Life Technologies, Carlsbad, CA). Nested PCR was carried out with 2 μl of the first round PCR product for 25 cycles in the same conditions as the first PCR. The nested PCR products were analyzed on 2% agarose gel stained with ethidium bromide.

The PCR product was purified using the Amicon Microcon-PCR centrifugal filter devices (Millipore Corporation, Billerica, MA) according to manifacturer’s instructions. Sequencing of both strands was performed with the same primers used in nested PCR using the BigDye 1.1 terminator kit (Applied Biosystems, Foster City, CA). Sequencing reactions were run on an automated DNA sequencer (ABI 310, Applied Biosystems). Sequences were compared with HEV reference isolates (https://blast.ncbi.nlm.nih.gov/Blast.cgi) using CLUSTAL X [29] and MEGA 4.1 [30].

## Competing interests

The authors declare that they have no competing interests.

## Authors’ contributions

PC and EM carried out serological assays; GP carried out Real-Time PCR; ST carried out sequencing and phylogenetic analysis; AC participated in data analysis and drafting the manuscript; SD, CV and AH participated in data analysis; RB contributed to the revision of the paper critically for important intellectual content; ARC had been involved in conception and design of the study, data analysis and interpretation and drafting the manuscript. All the authors had given final approval of the version to be published.
